# Supporting graduate medical trainees through parental leave: Best practices for program success

**DOI:** 10.1002/jhm.70386

**Published:** 2026-07-01

**Authors:** Jessica E. Zimo, Sarah E. Yentz, Maria E. Theodorou

**Affiliations:** ^1^ Internal Medicine Residency Program, Assistant Professor of Medicine, Division of General Internal Medicine, Department of Medicine Northwestern University Feinberg School of Medicine Chicago Illinois USA; ^2^ Internal Medicine Residency Program, Clinical Associate Professor of Medicine, Division of Hematology‐Oncology, Department of Medicine University of Michigan Ann Arbor Michigan USA; ^3^ Internal Medicine Residency Program, Division of Hospital Medicine, Department of Medicine Northwestern University Feinberg School of Medicine Chicago Illinois USA

## Abstract

Becoming a parent during medical training poses unique challenges. Programs and institutions vary widely in the time away from training and support offered in the peripartum period. Here, we offer four key strategies to support graduate medical trainees through parental leave and upon return to work: program specific policy development, transparent dissemination of policies, flexible peripartum elective offerings, and support of workplace lactation if desired by the trainee. Proactive support from program leadership before, during, and after parental leave can greatly improve trainee wellbeing and help establish a physician workforce well‐equipped to balance the competing priorities experienced by practicing physicians.

## INTRODUCTION

Becoming a parent during medical training poses unique challenges. Trainee health and wellbeing are paramount yet must be balanced with meeting institutional and accreditation body training requirements. Parental leave policies for medical trainees are primarily governed by the Accreditation Council for Graduate Medical Education (ACGME). As of 2022, ACGME Institutional Requirements mandate that all programs offer at least 6 weeks of paid medical, parental, or caregiver leave.^1^ The American Medical Association (AMA) recommends a more generous policy of 12 weeks.^2^ The ACGME Common Program Requirements for Residency mandate that resident and fellow parents be offered “an appropriate length of absence” for parental leave and that “policies must be implemented without fear of negative consequences for the resident.^3^” The spirit of these recommendations may, however, conflict with rigid specifications for cumulative time away from training permitted to remain board‐eligible in each medical specialty.

Disparities exist among specialties and by trainee gender. A 2020 analysis showed that plastic surgery and obstetrics/gynecology programs offered longer leaves up to 12 weeks′ duration, whereas the national median was only 5 weeks.^4^ In a 2024 study of over 5000 surgical residents, male residents reported pregnancies twice as often as female residents (22.3% vs. 10.2%).^5^ This gender disparity is alarming; despite facing equal clinical demands, the decision to have children is unequal.

Evidence suggests this is driven more by training environment culture than by trainee preference. In a cross‐sectional, observational survey of nearly 900 physician mothers across specialties, 32.3% delayed childbearing to complete training.^6^ In another survey, 61% of partnered female residents reported delaying childbearing during residency due to a lack of supportive parental leave policies. Only 38% were satisfied with their decision to delay parenting.^7^ As medical school admissions grow increasingly competitive, a rising number of applicants take gap years—58% in 2012 up to 65% in 2020.^8^ This has led to older medical school matriculants, with 57.4% aged 23–25 in 2024 compared to 51.9% in 2020.^9^ This has substantial implications, as rates of both infertility^10^ and adverse pregnancy outcomes^11^ rise with age.

While work‐life integration in training has improved over the past decade, it remains an intense and demanding period. As the average age of trainees rises, more individuals may choose to have children during training. To support trainees in making this choice, training programs must plan for parental leaves and facilitate smooth transitions back to clinical duties. Here, we highlight the benefits, barriers, and strategies to facilitate successful parental leaves in medical training programs.

### Benefits

At the program level, the duration of parental leave represents a critical opportunity to support trainee parents. In a study of over 200 female residents, when compared with residents with shorter maternity leaves, residents with ≥8 weeks of leave experienced less postpartum depression, breastfed longer, and were more satisfied with resident parenthood.^12^ Postpartum depression causes maternal suffering and diminished functioning and increases risk of impaired emotional, social, and cognitive development in children.^7^ Female residents are already at relatively increased risk for postpartum depression with reported rates ranging from 15% to 50%^12^, ^13^, ^14^, ^15^ compared to 13% in the general population.^16^

Prolonged breastfeeding has substantial health benefits. Infants that are exclusively breastfed for 6 months have a reduced risk of lower respiratory tract infections, severe diarrhea, otitis media, and childhood obesity. For mothers, breastfeeding beyond 12 months has been linked to lower rates of type 2 diabetes, hypertension, breast cancer, and ovarian cancer.^17^ Despite this, 37% of residents^18^ and 58% of trainee surgeons^19^ report being unable to meet their lactation goals.

### Barriers

A major barrier for trainee parents is inconsistent visibility and comprehensiveness of program leave policies. A 2025 survey of residents across four specialties showed substantial differences in policy and perception of pregnancy during training that impacted resident quality of life.^20^ Variable policies can lead to inconsistent implementation and utilization of trainee benefits. Furthermore, inadequate explanation of the interplay between parental leave policy and specialty‐specific allowances for time away from training may limit resident understanding of the potential implication (or lack thereof) of taking parental leave of a given duration on board eligibility.

Limited transparency around parental leave policies perpetuates a taboo which may dissuade trainees from childbearing during training. A 2024 study of orthopedic surgery residencies found that only 6% of programs had publicly accessible parental leave policies; many required residents to directly request policy details, a barrier which could perpetuate gender disparity.^21^ In internal medicine residency programs, 86% of program directors reported having a written parental leave policy, but only 43% had program‐specific policies. Program‐level policies are considerably more likely than institutional policies to address scheduling, peer coverage, and training extension requirements.^22^

Barriers to standardized parental leave policies exist at the program and trainee level. In a survey of neurology residency program directors, limiting factors included concerns about staffing, equity, and perceived clinical training disruptions.^23^ Despite the health benefits of parental leaves ≥8 weeks, financial constraints and fear of extending training deter many trainees from accepting the full duration of leave available.^12^

Upon returning to work, trainees face challenges of sleep disruptions and the logistical challenges of integrating childcare with clinical responsibilities. A national survey found that 63% of surgical trainees face difficulties arranging childcare, with the burden falling disproportionately on women.^18^ In a multispecialty survey, 72% of trainee parents reported childcare was “at least quite stressful,” and 68.6% reported it contributed to burnout.^24^ If lactation is desired, a lack of protected time, colleague support, and private, convenient locations to pump and store breastmilk hinder trainee success^17^, ^25^

### Suggested strategies

**Figure 1:**
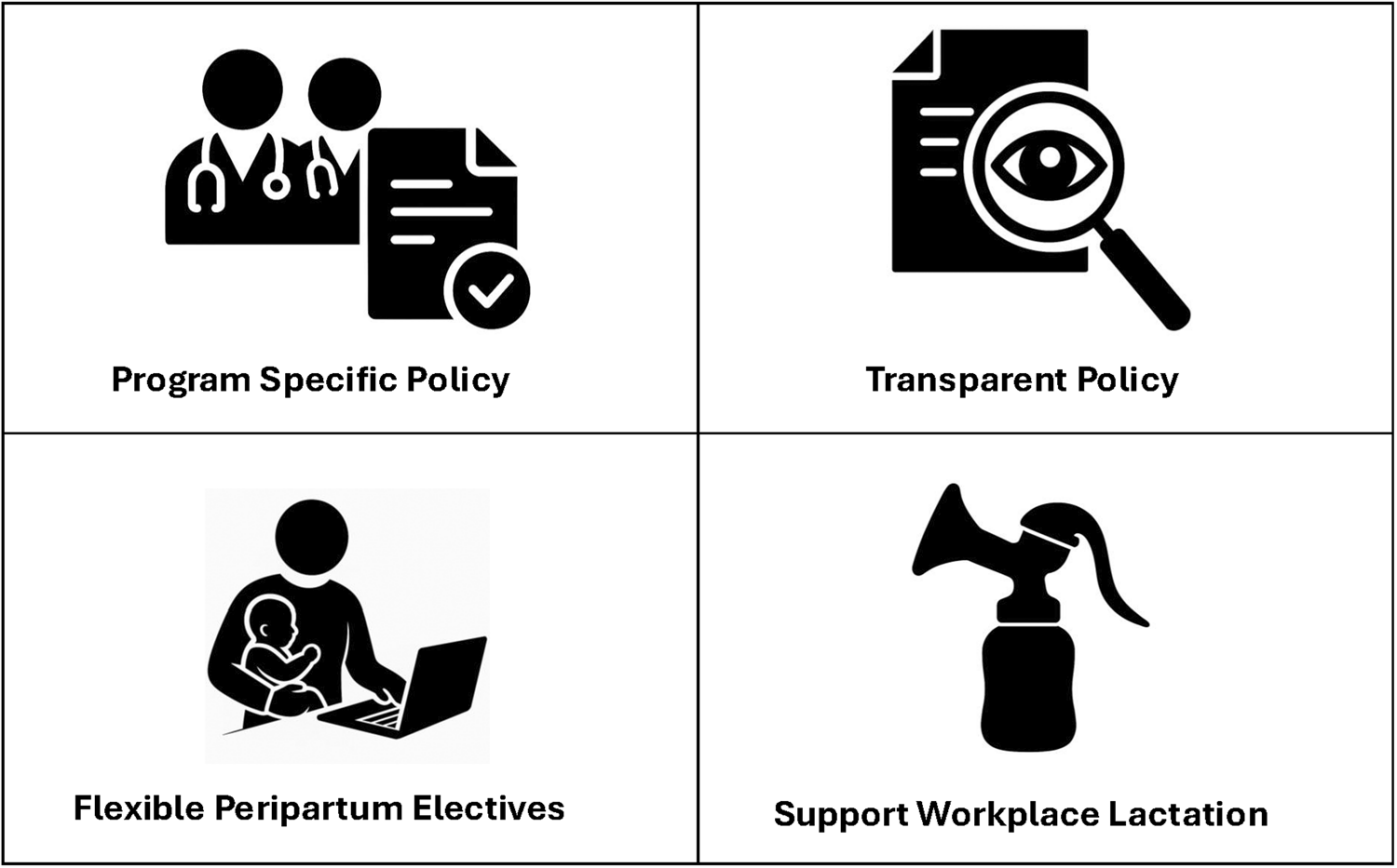
Key strategies to support graduate medical trainees through parental leave.

Creating standardized program‐specific policies can enhance the transparency and accessibility of parental leave and reduce the mental load of trainee parents (Figure 1). Programs should implement uniform policies meeting a standard of at least 8 weeks of leave for birthing parents and tailored to their own training requirements and staffing needs. Preemptively establishing strategies to address program‐specific staffing issues enables thoughtful and dynamic responses to parental leaves. Express inclusion of specialty‐specific requirements for board eligibility in parental leave policies and allows for shared decision making around the potential implications of parental leave on a trainee′s professional timeline. Specialty requirements change over time and are not uniform across all specialties. Some trainees may accept extension of training or delayed board certification to accommodate longer parental leave if permitted, while others may prioritize completion of training and board certification so as not to delay subspecialty training or entrance into the physician workforce.

Making policies readily available to prospective and current trainees can inform trainee decisions about childbearing and establish clear coverage expectations, alleviating tension amongst trainees. Creating standard practices and utilizing proactive schedule adjustments over sole reliance on backup call systems facilitate predictable and equitable coverage plans. Publicizing policies that are implemented by designated program leaders may reduce the potential guilt of trainee parents and encourage them to take adequate parental leave.^26^

Educational resources for new parents and peer mentorship can help trainees navigate returning to work. Birthing parents‐to‐be should be educated about the benefits of taking at least 8 weeks of parental leave. Hearing the experience of a peer who recently experienced pregnancy during training can normalize parent needs and encourage adequate durations of leave.

Dedicated curriculum can also promote a smooth return to work. Self‐directed remote “Parenting Electives” are widespread among pediatric residencies.^27^ Despite the broad clinical relevance of childbearing, similar offerings are uncommon in other specialties. At our internal medicine programs, we developed “Postpartum Electives” addressing reproductive health topics for internists. These rotations utilize a hybrid structure wherein residents learn about peri‐partum healthcare while spending time with their new child.

When residents return to work, program leaders should proactively reduce barriers to lactation. Examples include modifying clinical schedules, offering to communicate with supervisors, and ensuring access to lactation spaces. We recommend that trainee lactation spaces be located close to the clinical work environment and include a workstation. While working during a lactation break should not be required, trainees may feel more comfortable if they have the option to remain connected to patient care during these times.

## CONCLUSION

Medical training is a pivotal phase in a physician′s career, and the lack of program‐specific parental leave policies imposes unnecessary burdens on trainees. It is essential that training programs implement standardized, accessible, and supportive leave policies and plans for return to work that allow trainees to thrive professionally and personally. Proactive support from program leadership before, during, and after parental leave can greatly improve trainee wellbeing. Training programs must prioritize parental leave as a necessary step toward a sustainable, equitable future in medicine.

## CONFLICT OF INTEREST STATEMENT

The authors declare no conflicts of interest.

## Data Availability

Data sharing is not applicable to this article as no datasets were generated or analyzed during the current study.
